# Perceptions of hypertension treatment among patients with and without diabetes

**DOI:** 10.1186/1471-2296-13-24

**Published:** 2012-03-26

**Authors:** Heymann Anthony, Liora Valinsky, Zucker Inbar, Chodick Gabriel, Shalev Varda

**Affiliations:** 1 Medical division, Maccabi Healthcare Services, Tel Aviv, Maccabi, Israel; 2 Sackler Faculty of Medicine, Tel Aviv University, Tel Aviv, Maccabi, Israel; 3 Ministry of Health, Jerusalem, Israel

**Keywords:** Hypertension, Diabetes Mellitus, Attitude to health, Comprehension, Patient Compliance

## Abstract

**Background:**

Despite the availability of a wide selection of effective antihypertensive treatments and the existence of clear treatment guidelines, many patients with hypertension do not have controlled blood pressure. We conducted a qualitative study to explore beliefs and perceptions regarding hypertension and gain an understanding of barriers to treatment among patients with and without diabetes.

**Methods:**

Ten focus groups were held for patients with hypertension in three age ranges, with and without diabetes. The topic guides for the groups were: What will determine your future health status? What do you understand by "raised blood pressure"? How should one go about treating raised blood pressure?

**Results:**

People with hypertension tend to see hypertension not as a disease but as a risk factor for myocardial infarction or stroke. They do not view it as a continuous, degenerative process of damage to the vascular system, but rather as a binary risk process, within which you can either be a winner (not become ill) or a loser. This makes non-adherence to treatment a gamble with a potential positive outcome. Patients with diabetes are more likely to accept hypertension as a chronic illness with minor impact on their routine, and less important than their diabetes. Most participants overestimated the effect of stress as a causative factor believing that a reduction in levels of stress is the most important treatment modality. Many believe they "know their bodies" and are able to control their blood pressure. Patients without diabetes were most likely to adopt a treatment which is a compromise between their physician's suggestions and their own understanding of hypertension.

**Conclusion:**

Patient denial and non-adherence to hypertension treatment is a prevalent phenomenon reflecting a conscious choice made by the patient, based on his knowledge and perceptions regarding the medical condition and its treatment. There is a need to change perception of hypertension from a gamble to a disease process. Changing the message from the existing one of "silent killer" to one that depicts hypertension as a manageable disease process may have the potential to significantly increase adherence rates.

## Background

Hypertension is a leading cause of cardiovascular morbidity and mortality. In 2006 in the Unites States, hypertension was documented as the primary cause of death in over 56,000 deaths and as a contributing factor in an additional 250,000 deaths out of the 2.4 million deaths reported for that year [1]. Hypertension is a common chronic disease, estimated to affect over 29% of the adult population as reported in The National Health and Nutrition Examination Survey (NHANES) 1988-1994 and 1999-2008, which represents a probability sample of the US civilian population [2]. The high prevalence of hypertension together with its deleterious effect on health make it a major public health problem; In a recent report the Institute of Medicine called hypertension "a neglected disease that is often ignored by the general public and underappreciated by the medical community" [3]. Despite the availability of a broad selection of effective antihypertensive treatments and the existence of clear treatment guidelines, approximately one third of hypertensive patients do not have their blood pressure controlled. Hypertension is a particularly important issue among people with diabetes as it is a significant contributor to complications of diabetes such as kidney failure and retinopathy as well as increasing cardiovascular mortality [4].

In a study conducted between September 2005 and March 2006 in 26 countries, overall blood pressure (BP) control rate was 33.6% in men and 30.6% in women (P < 0.0001) and was lower in diabetic as compared with non-diabetic patients [5]. Factors affecting BP control can be identified at the level of the patient, the health provider and the health system [6]. One of the major problems identified in the literature is poor adherence to medication among patients, which has been reported to range between 43% to 88% [7]. If optimal blood pressure treatment is seen as a combination of medications and lifestyle modifications, the level of adherence to recommendations is even lower [8]. Hypertension treatment often requires a combination of medications. Patients may choose to take all or some of the prescribed medications resulting in varying levels of non-adherence [9].

A recent study of 4783 patients who participated in studies whose dosing histories were available through electronic monitoring showed that among patients prescribed once a day antihypertensive drugs, non-persistence is the major problem: half of the patients stopped treatment within a year and 48% admitted to at least one "drug holiday" (not adhering to medications for a short period of time) a year [10]. Patient adherence to prescribed treatment is a complex phenomenon, known to be influenced by patient knowledge and beliefs about his disease and about the benefits and risks of its treatments [11]. Many reasons have been cited which may prevent physicians from treating hypertension effectively. Among those cited is failure to adhere to treatment guidelines, problems in physician-patient communication, a lack of training and skills in lifestyle counseling and work under severe time constraints [6,12]. The difficulty of accepting and coping with a diagnosis of hypertension has been described in a number of studies. In one study as many as 66% of patients had difficulty accepting the diagnosis of hypertension [13]. Patient denial, mistrust and hopelessness regarding hypertension have been reported in a small qualitative study [14]. Patients with diabetes usually adhere to medication regimes for the treatment of hypertension and are more likely to adhere to these than dietary and lifestyle modifications, but are more willing to do so if their physician advises them to [15,16]. This has become even more important in view of the positive effects of the DASH diet in relation to disease progress in diabetes as well as hypertension [17].

Possible problems at the health system level include insufficient availability of medical services, cost of medications and poor patient adherence. The focus of this study is on the reasons that patients do not adhere, or adhere only partially to medication and lifestyle regimes.

In spite of the fact that hypertension is easy to identify, and that there are a variety of pharmaceutical and non-pharmaceutical (such as salt reduction, dietary modification and weight loss) evidence-based treatment options available, we are failing miserably at hypertension control. If we can gain an understanding of why people with hypertension who have been advised what they need to do and provided with prescriptions for inexpensive and readily available medications are not adhering to treatment, we will be able to modify the ways in which we communicate with patients in order to increase adherence. The aim of this study was to gain a deeper understanding of the beliefs, attitudes and coping mechanisms of patients with hypertension. We also aimed to examine the differences between males and females, and between younger and older age groups, and the differences between those who had an additional, more severe chronic disease; diabetes, and those who do not, in regards to hypertension and its treatment. Focus group methodology was chosen in order to generate hypotheses for further research. Better understanding of these perceptions can help both the physicians and the health system to develop effective communication strategies to increase patient adherence to hypertensive treatment.

## Methods

### Setting

This qualitative study was conducted in a large publicly financed health maintenance organization with 1.9 million members. Membership is open to all sectors in the general population. A computerized medical record is used and complete clinical data have been collected in a central database since 1995. Based on these data, diabetes and hypertension registries have been developed. These are built using algorithms that identify members who have the disease, and are continually validated by physicians. There are over 250,000 people in the hypertension registry, of which approximately 60,000 are also in the diabetes registry. This study was approved by our institutional review board. Informed consent was not required.

### Population selection

Participants in the patient focus-groups were randomly selected from the hypertension registry. Randomization was stratified by age, gender and diabetes status, as these factors were expected to influence patient's attitudes regarding hypertension and their mode of management. We produced a list of 150 potential participants, and patients were contacted by phone and asked to participate in focus groups on a health issue. Patients were contacted until the required number was recruited. We did not ask the reasons for non participation. Participants were not aware of the topic of the focus group. They were invited to the focus groups by an external expert provider, and were compensated for their time. This was designed to achieve diverse opinions organized by age and gender. The study design was based on several assumptions: That men and women would discuss health issues more freely in separate groups, that attitudes towards hypertension would differ between younger and older patients, and that those who have an additional chronic disease, which is perceived as more severe, may have different attitudes towards chronic disease in general, and hypertension in particular. We therefore conducted separate focus groups were for men and women with hypertension in three age ranges: 41-50, 51-60, 61-70 years (a total of six groups), and for men and women with hypertension and diabetes in two age ranges: 51-60, 61-70 years (a total of four groups). A lower cutoff age of 40 was selected as the majority of patients in the hypertension registry are aged 40 and over, and we felt that we should address the issue in a relatively homogenous group, as it may be that treating a much younger hypertensive is a totally different issue.

### Focus-group preparation

Topic guides for the patient groups were developed by a multidisciplinary team which consisted of a health promotion expert, primary care physicians, nurses and social psychologists. The topic guide for the patient's focus group covered the following domains:

1) What will determine your future health status (Probe: current health situation, lifestyle, luck, and genetics).

2) What do you understand by "raised blood pressure"(Probe: causes, risks, treatments, personal and family experiences, impact on function).

3) How should one go about treating raised blood pressure? (Probe: difficulties, side effects of treatments including impact on sexual function, coping mechanisms, attitudes of primary care medical team).

### Focus-group sessions

These were facilitated by one of two psychologists. The male psychologist ran the male groups and the female psychologist the other groups. Each session included 7-10 participants and lasted 1.5-2 hours. Sessions began with brief introductions followed by a short discussion on health in general, and how participants view their own health. Hypertension was introduced as the subject of the study using open questions included in the topic guide. New topics were introduced after everyone wishing to express their opinions had finished doing so. Having obtained consent discussions were audio taped and transcribed verbatim, in addition to documentation of the discussion by the psychologists during the sessions. The venue selected for the sessions was not identified with any health organization. Participants were not informed at any point who is conducting the study, what the aim of the study is, or how participants were selected.

### Data analysis

The same or following day the written notes were typed and compared with the audiotape. An overview grid was constructed which provided a descriptive summary with topic headings on one axis and focus group identifiers on the other [18]. The strength of consensus for each topic was assessed. The break characteristics used to differentiate subsets of the target population were age, patients who also had diabetes and patient gender. The material collected in the focus groups was analyzed for recurrent themes separately by several experts, and cross referenced to identify incidents of misidentification. Central categories were identified within the material, and documented according to the pre-identified research questions. Deviant cases were discussed. The summaries were then reviewed again, to verify, adjust and re-allocate categories. Lastly, the relationship between the categories and the dynamics between them were presented as power fields within which the research issue is examined [19]. The number of groups selected reflects the minimal number that is necessary to represent the relevant populations. The number of groups and population distribution within each group were determined by the minimal number required to obtain an appropriate picture of the research issues as presented in the research questions. The number of groups necessary was determined by the variety and the size of each of the sub-populations in the hypertension registry. In this way we determined that our samples were of sufficient size, number and variety to ensure a reasonable level of information saturation.

## Results

We conducted ten focus groups, with 86 patients: 42 men and 44 women Of these, 37 participants had both hypertension and diabetes. Most of the participants initially denied having "hypertension", and when coaxed, all agreed that they had a "blood pressure problem" or that occasionally their blood pressure was higher than normal. The first issue that was brought up was the issue of health in general, and future health in particular. The word/phrase codes that were dominant regarding the question of future health status were "stress" and "lifestyle". Participants did not, in general, bring up the issue of chronic illness in general, or diabetes in particular. The issue of hypertension was not mentioned by any participant. Regarding the question of "What do you understand by raised blood pressure?" the highest ranking code by far was "stress". Codes relating to chance/fate were also dominant in this part of the discussion as were health outcomes such as "stroke heart attack". The dominant codes that were recorded regarding the treatment question were "medications", "stress". Side effects came low in the ranking. The focus groups were divided according to gender because our assumption was that the medications' effect on sexual function would be an important theme. This turned out not to be the case.

### Patients' perception of hypertension

Patients with diabetes regard hypertension as just another health problem: *"It is a disease like any other disease and it has treatment... pills and diet"*

All patient groups expressed the view that hypertension is less of a disease and more of a risk factor because of its potential for dramatic events: "*It is a silent disease called 'the silent killer'... it is very dangerous mainly since you do not feel it and it causes severe damage... it kills". "With hypertension you feel healthy... there are no ill effects... you take a pill and you can forget. In diabetes it is a different story. It's a different feeling and demands my full attention... you can't forget it."*

Most patients in both groups avoid calling hypertension a disease, because of its asymptomatic nature. *"It is a limitation but not a disease because you feel healthy"*

However patients with diabetes who already have a chronic disease label, are willing to accept that hypertension is a chronic condition. Patients without diabetes generally found it difficult to accept that hypertension is a chronic condition and see it as a physiological reaction to stress.

"I see it as a problem and not a disease... "

"Blood pressure is a control light of the body.... When it is high it is a sign that something is wrong and you need to treat it"

A recurrent theme in the discussions was that the patients attribute personal stress as a significant cause of their elevated blood pressure and as a result they view stress reduction as an effective way to control it.

"I learned not to get angry and not have my feelings hurt but when I get angry even about something small I feel how my blood pressure rises"

The discussions exposed gaps in the patient understanding of the hypertension, its possible consequences and the factors affecting it. This was clearly expressed by the diabetic patients who reported that the explanation they received regarding diabetes was much more comprehensive than the explanation received regarding hypertension. Both groups felt that hypertension does not cause immediate damage, and is in fact similar to a time bomb, which can either explode or not. Diabetic patients specifically stated that while diabetes is an insidious disease that causes immediate damage, hypertension is more like a risk factor for future events.

### Patient modes of management

Patients presented a few modes of coping with hypertension, depending on their individualized perceptions regarding the disease and its treatments:

### Full adherence

A minority of the participants followed a combination of adherence to medications and "drastic" lifestyle modifications

### Compromise

This mode reflects a compromise between the 'ideal treatment' and 'palatable' treatment which is reached by balancing the perceived importance of the treatment and the degree of disruption to their desired lifestyle that this treatment involves. These patients found it easier to comply with taking pills than to change their diet, quit smoking or exercise.

*"... so I take pills, sometimes eat healthy, swim a little... I am not willing to invest everything in health, hypertension will not disrupt my lifestyle"*. It seems that even the random occurrences of forgetting to take a pill or to renew the prescription on time comes from a decision to not to put too much thought and effort into the treatment.

Self-adjustment: These patients modulate their doctor's orders. They believe they "know their bodies" and hence have a better ability to better control their blood pressure. They tend to overestimate the effect stress carries on blood pressure: *"I learned to control stress and according to that I lower the dosage of my pills or increase it"*. Out of the treatment modalities, they tend to prefer lifestyle modifications over medications which they often perceive as a mechanistic and impersonal approach.

No treatment: There are patients who not treat their blood pressure, either because they deny having elevated blood pressure or they deny the need to treat it. By denying the disease they avoid facing the anxiety that is associated with the diagnosis.

"If there is high blood pressure, I do not want to know... thinking of that makes me anxious... I am healthy because I feel healthy"

There are patients who stopped taking anti-hypertensive medication because they view hypertension as a temporary state and not as a lifetime diagnosis. Some stopped their treatment because they had an adverse reaction and for them that was the only negative manifestation of their blood pressure: *"The doctor found that I have blood pressure. The pill caused heartburn so I stopped taking it."*

Patients who also had diabetes described two different coping strategies for dealing with their hypertension. Some saw hypertension as a separate and less important part of their routine and represented by the "compromise group": "*Hypertension is a small issue for me. I take pills because they are part of my life today but no more than that. Most of my energy is invested in diabetes." *Others dealt with both conditions using a holistic "Full adherence" approach: *" The diabetes forces me... it screams at me and in order to deal with it I need to be strict. So there is no separation of hypertension from diabetes... you live a more healthy life"*. Another patient stated: *" If you want to live a long life you need to be active, live a strictly healthy lifestyle and deal with all disease. So that diabetes, cholesterol and hypertension are all in the same basket*."

Table 1 summarizes the main differences between those hypertensive patients with and without diabetes.

**Table 1:** Summary of focus group findings

Issue	Hypertensive patients with diabetes	Hypertensive patients without diabetes
Hypertension as a disease	Accept that hypertension is a chronic disease	See hypertension as a temporary physiological reaction to stress

Importance of hypertension	Hypertension has the potential to cause major damage but minor in comparison with diabetes	Hypertension has the potential to cause major damage

Impact of disease	As opposed to diabetes, which cause cumulative damage hypertension does no immediate damage and is a long term risk factor for an outcome that may or may not happen	Hypertension does no immediate damage and is a long term risk factor for an outcome that may or may not happen

Coping mechanisms	Treatment is easy compared to care of diabetes and becomes part of their routine	Treatment can be overwhelming and there are several different coping mechanisms

Impact of medication on sexual function	Effect is acknowledged but thought to be minor compared with diabetes	None of the male participants acknowledged any impact

## Discussion

Listening to patients as "experts" is essential in addressing barriers to adherence with medical advice [20]. In this study we explored some of the attitudes and perceptions held by hypertensive patients with and without diabetes, regarding hypertension and its management. When comparing between the two groups, we found several differences. Patients with diabetes are more accepting of the fact that hypertension is a chronic condition that needs to be treated systematically for the duration. This may be related to their having accepted their role as a patient with a chronic illness in relation to diabetes, and also to their being, as a rule, older than participants without diabetes, and therefore more open to the deterioration of health associated with ageing. Patients with diabetes saw hypertension treatment as non-burdensome in comparison with the rigors of caring for their diabetes. There was one major issue, however, which was common to all participants: the view that hypertension is a gamble, which can either have or not have serious effects in the future. The well-established positioning of hypertension as the 'silent killer' may have captured the public imagination but it also may have a distinct downside: it downplays the chronic damage caused by hypertension. This was clearly expressed in the focus group discussions by one diabetic patient who visualized hypertension as a snake that can quietly and suddenly strike you and diabetes as a worm that eats you slowly. As a result, patients view non-adherence with treatment as a type of gamble which may be successful so long as no catastrophe happens. This finding adds an explanatory layer to findings from a recent qualitative study that found that patients see hypertension as a serious disease but do not adhere to treatment [21]. We can only speculate that if patients were better informed about the chronic damage inevitably caused by exposure to elevated blood pressure, then they would be more inclined to adhere to their advised treatment, since fear of complications was shown to be a solid reason for treatment adherence [22].

The focus group discussions showed that in general most patients are aware of the recommendations to combine medical treatment with lifestyle modification. However, only a minority of them adhered to these recommendations. More commonly, patients choose to combine only some of the recommended treatment elements (e.g. pharmacotherapy, dietary salt reduction etc.) and only to a certain extent. Patients' selection of a mode of management is influenced on the one hand by their perceptions regarding the dangers of hypertension and the necessity of treatment and by their personal experiences and beliefs about the various treatment modalities. The lay perception of stress as a dominant cause of hypertension can be found in many cultures and supports the tendency of these patients to abandon their pharmacotherapy and adopt less established treatment modalities such as meditation [21,22], or, as we found, to take their medications only when they are feeling stressed.

At the other end of the spectrum there are patients who focus on adhering to medications, and view it as a sufficiently appropriate substitute to the more demanding and time consuming recommended lifestyle changes.

Finding better ways of communicating with patients regarding hypertension treatment, and particularly in relation to lifestyle changes is essential [23,24].

A possible strategy to improve hypertension management is public education both for the general population and people with hypertension [25]. Insights obtained from these focus groups specifically regarding misconceptions about potential damage from hypertension should be further investigated and used to formulate future educational messages.

Strengths and limitations: Focus group study design is used to generate a hypothesis. These focus groups enabled us to gain a deeper understanding of patients' perceptions of the term "hypertension" and of possible sources of miscommunication in the clinical setting, due to the open discussions that occurred in the groups. Holding separate focus groups for patients of different ages and genders can expose a wide range of views that are not usually expressed in the routine patient-doctor encounter. One of the major limitations of the study is that participants in the focus groups do not form a representative sample of the population. Our research design did not allow us to identify ethnic or socioeconomic differences between those who agreed to participate in the focus groups and those that declined. More importantly the mere participation in focus groups select a population with higher health awareness so it is expected that the group discussions gave an underestimated picture of patients' non adherence. It may be that the authors (ADH and LV) who were present during the discussions which formulated the topic guides may have unduly influenced their formulation.

## Conclusion

This study suggests that patients as a rule do not see hypertension as a chronic disease requiring adherence to treatment recommendations, but rather as a health condition mainly related to stress, that may actually have no consequences if left untreated. Although we found little difference between age and gender groups, patients with diabetes have a different view of hypertension than those who do not. Patient denial and non-adherence with hypertension treatment seems to be a prevalent phenomenon reflecting a conscious active choice made by the patient, based on his knowledge and perceptions regarding the medical condition and its treatment. Specifically referring to hypertension as an illness, highlighting the chronic damage caused by exposure to high values of blood pressure and providing patients with information about the importance of the various treatment modalities in a way that would address their individual reservations may improve control of both diabetes and hypertension.

## Competing interests

The authors declare that they have no competing interests.

## Authors' contributions

The study was conceived by ADH, VS and LV. ADH and LV ran the focus groups and edited the manuscript. IZ wrote the manuscript. VS and GH helped with the analysis, reviewed and edited the manuscript. All authors read and approved the final manuscript.

## Funding

There was no external funding for this research.

## Pre-publication history

The pre-publication history for this paper can be accessed here:

http://www.biomedcentral.com/1471-2296/13/24/prepub
